# Evolutionary profiles for protein fitness prediction

**DOI:** 10.1093/bioinformatics/btag525

**Published:** 2026-07-20

**Authors:** Xiaoran Jiao, Shengdong Lin, Jigang Fan, Zhanming Liang, Weian Mao, Hao Chen, Chunhua Shen

**Affiliations:** Computer Science and Technology, Zhejiang University, Hangzhou, 310058, China; School of Information Science and Engineering, East China University of Science and Technology, Shanghai, 200237, China; Center for Data Science, Peking University, Beijing, 100871, China; College of Atmospheric Sciences, Chengdu University of Information Technology, Chengdu, 610225, China; Computer Science and Artificial Intelligence Laboratory (CSAIL), Massachusetts Institute of Technology, Cambridge, MA 02139, United States; Computer Science and Technology, Zhejiang University, Hangzhou, 310058, China; Computer Science and Technology, Zhejiang University, Hangzhou, 310058, China

## Abstract

**Motivation:**

Predicting the fitness impact of mutations is central to protein engineering but constrained by limited assays relative to the size of sequence space. Protein language models (pLMs) trained with masked language modeling (MLM) exhibit strong zero-shot fitness prediction; we provide an interpretive lens by regarding natural evolution as implicit reward maximization and MLM as inverse reinforcement learning (IRL), in which extant sequences act as expert demonstrations and pLM log-odds serve as fitness estimates.

**Results:**

Building on this perspective, we introduce EvoIF, a lightweight model that integrates two complementary sources of evolutionary signal: (i) evolutionary profiles from retrieved homologs and (ii) inverse folding (IF) profiles distilled from IF logits. EvoIF fuses sequence–structure representations with these profiles via a compact transition block, yielding calibrated probabilities for log-odds scoring. On ProteinGym (217 mutational assays; >2.5M mutants), EvoIF and its MSA-enabled variant achieve competitive performance while using only 0.15% of the training data and fewer parameters than recent large models. Ablations confirm that evolutionary and IF profiles are complementary, improving robustness across function types, MSA depths, taxa, and mutation depths.

**Availability and implementation:**

Code is archived on Zenodo at https://doi.org/10.5281/zenodo.20139484.

## 1 Introduction

Protein evolution is driven by selective pressure: mutations that preserve or enhance function are preferentially retained, whereas deleterious ones are eliminated (Göbel *et al.* 1994). The success of a protein variant within this evolutionary landscape is quantified by its fitness, a measure of its functional viability, and contribution to an organism’s survival. Mapping this sequence–function relationship, commonly referred to as the fitness landscape, is therefore a central challenge in molecular biology. Accurate prediction of mutational fitness forms the foundation of rational protein design (Romero and Arnold 2009, Notin *et al.* 2024), enabling the engineering of enzymes with enhanced catalytic efficiency, antibodies with improved affinity, and biologics with increased stability. In practice, fitness prediction is often used to prioritize candidate mutations for experimental validation when testing capacity is limited. It therefore provides a computational route for exploring large mutational spaces in therapeutics, materials science, and sustainability.

Protein fitness prediction is constrained by the scarcity of experimental measurements relative to the vastness of protein space (Biswas *et al.* 2021). Consequently, self-supervised methods for protein representation learning have become essential for protein fitness prediction (Hopf *et al.* 2017, Meier *et al.* 2021, Zhang *et al.* 2024). Recently, protein language models (pLMs) including ESM series (Rives *et al.* 2021, Lin *et al.* 2023) and their structure-informed variants (Hsu *et al.* 2022), trained through masked language modeling (MLM), have demonstrated remarkable zero-shot capabilities in protein fitness prediction (Notin *et al.* 2023). These models can predict the impact of mutations on protein function without additional training specific to particular protein families, sometimes achieving performance comparable to specially trained models. Current state-of-the-art approaches, including AIDO-Protein-RAG (Li *et al.* 2024a) and VenusREM (Tan *et al.* 2024), further boost performance by integrating homologous sequences as evolutionary context.

Although the encouraging results mentioned above, current methods still confronted with several substantial challenges:

Issue 1. Most pLMs are trained using the MLM task, yet there is still a lack of a reasonable explanation for why MLM can serve as a proxy task for protein fitness prediction.

Issue 2. Current approaches tend to focus heavily on scaling model parameters and training data, yet the performance gain in protein fitness prediction remain marginal (Fig. S2, available as supplementary data at *Bioinformatics* online). Moreover, the computational requirements for pre-training and further fine-tuning such large-scale models can be extremely high, which may restrict their practical applicability in resource-constrained settings.

**Figure 1:**
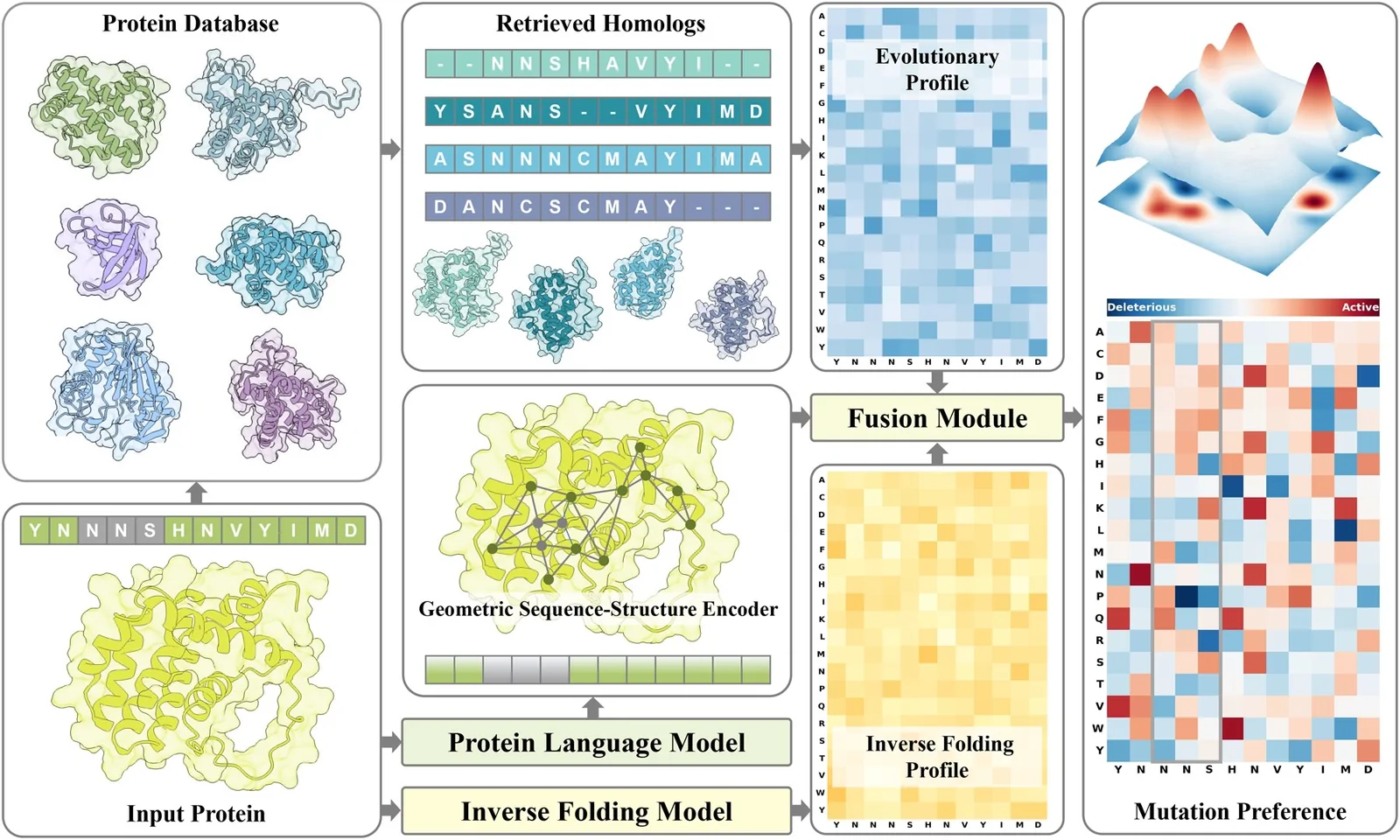
Overview of the proposed EvoIF framework, integrating sequence–structure representations with evolutionary and inverse folding profiles for zero-shot protein fitness prediction.

Issue 3. Existing models have not fully considered the comprehensive modeling of protein evolutionary information. For sequence evolution information, researchers have applied multiple sequence alignment (MSA) (Edgar and Batzoglou 2006) for modeling. In contrast, inverse folding (IF) (Hsu *et al.* 2022) has been developed to model cross-family structural evolutionary information. Notably, MSA relies solely on sequences, while IF depends solely on structure. Therefore, for a protein with both sequence and structure, it is natural to construct a comprehensive evolutionary model that incorporates both its sequence and structural information. However, this aspect remains underexplored. The majority of research treats structure merely as part of protein representation, overlooking the evolutionary information embedded within it (see Fig. 1).

To address the issues mentioned above, this article makes the following contributions:

1. We first propose that protein evolution can be viewed as an implicit reward-maximization process in which natural selection acts as an expert that iteratively selects high-fitness sequences; the resulting extant sequences therefore constitute an expert demonstration set. From this perspective, MLM pre-training aligns with inverse reinforcement learning (IRL) (Ng and Russell 2000): recover the latent reward (fitness) from the observed expert’s behaviors (protein sequences). We show that the maximum-likelihood objective of MLM coincides with the maximum-entropy IRL loss (Ziebart *et al.* 2008); accordingly, the log-odds ratio produced by a pLM provides an estimate of protein fitness.
2. We explicitly incorporate sequence evolutionary information from homologous sequences of the same family into the model. This information is obtained through sequence similarity searches (Edgar and Batzoglou 2006), or structure similarity searches such as Foldseek (van Kempen *et al.* 2024), to identify the most closely related sequences within the same family. These sequences exhibit the most direct sequence or structure homology and have been shown to be beneficial for predicting protein fitness (Li *et al.* 2024a, Tan *et al.* 2024). By providing family-specific evolutionary context, these homologous sequences strengthen the profile-level signal used for fitness prediction.
3. Furthermore, we attempt to explicitly integrate cross-family structural evolutionary information into the model. While there has been extensive research on modeling sequence MSA, it is ultimately the 3D structure encoded by these sequences that determines protein function and activity. During protein evolution, accumulated mutations lead to corresponding structural changes, thereby driving fitness evolution (Shanker *et al.* 2024). The IF model can predict high-confidence amino acid sequences compatible with a given backbone structure, effectively performing the inverse task of structure prediction. Since it is trained on natural protein structures and sequences, it is capable of capturing the complex distribution patterns of protein sequences shaped by evolutionary dynamics. Recent studies (Shanker *et al.* 2024, Fei *et al.* 2025) suggest that the IF model tends to select amino acids similar to natural variants, indicating that it has internalized key structural–evolutionary couplings across families. Therefore, we treat the likelihood values provided by the IF model as a compact structural evolutionary profile and explicitly incorporate it into the model to provide cross-family evolutionary information.

In summary, we propose EvoIF, a lightweight network that combines (i) within-family evolutionary information from homologous sequence MSA retrieved through sequence or structure searches, and (ii) cross-family evolutionary information embedded in the IF likelihood values, together with its MSA-enabled variant, EvoIF-MSA. By effectively integrating evolutionary features from homologous sequences and cross-family structures, EvoIF offers a data-efficient solution: in the deep mutational scanning (DMS) (Fowler and Fields 2014) experiment of over 2.5 million mutants across 217 proteins in ProteinGym (Notin *et al.* 2023), it achieves competitive performance while requiring only 0.15% of the training data used by large-scale pLMs. Additional ablation studies demonstrate that these different dimensions of evolutionary information complement each other well and show strong robustness as training data is further reduced. Together, these results suggest that EvoIF is an efficient and robust network for modeling evolutionary information. EvoIF provides accurate protein evolutionary profiles, and due to its lightweight nature, it enables fine-tuning for specific proteins or tasks, offering broad benefits.

### 1.1 Related work

Protein fitness prediction methods differ mainly in how they represent evolutionary and structural constraints. Alignment-dependent methods such as EVE (Riesselman *et al.* 2018), GEMME (Laine *et al.* 2019), and DeepSequence (Frazer *et al.* 2021) model conservation and co-evolution from MSAs. Family-agnostic pLMs, including ESM-2 (Lin *et al.* 2023), ProGen2 XL (Nijkamp *et al.* 2023), and CARP-640M (Yang *et al.* 2024), support zero-shot mutation scoring from sequence likelihoods. Structure-aware and sequence–structure models, including ProteinMPNN (Dauparas *et al.* 2022), MIF (Yang *et al.* 2023), ESM-IF (Hsu *et al.* 2022), ProtSSN (Tan *et al.* 2025), SaProt (Su *et al.* 2024), S2F/S3F (Zhang *et al.* 2024), and SSEmb (Blaabjerg *et al.* 2024), incorporate geometric priors or structural representations. Retrieval-enhanced methods such as MSA Transformer (Rao *et al.* 2021), Tranception and TranceptEVE (Notin *et al.* 2022a, 2022b), VenusREM (Tan *et al.* 2024), and AIDO-Protein-RAG (Li *et al.* 2024a, Sun *et al.* 2024) further use family-specific or retrieved evolutionary context. These lines of work show that sequence likelihoods, structural constraints, and evolutionary context are all useful, but they are often modeled by separate modules, large backbones, or retrieval-heavy systems. EvoIF targets a complementary point in this design space: it keeps the backbone compact and fuses Foldseek-retrieved structural homolog profiles with structure-conditioned IF profiles for zero-shot log-odds scoring.

## 2 Method

As shown in Fig. 1, we present EvoIF, a data-efficient framework for protein fitness prediction that (i) encodes sequence–structure context with a lightweight sequence–structure backbone and (ii) injects evolutionary information through two compact profiles: an evolutionary profile and an IF profile. The fused probabilities enable zero-shot log-odds scoring consistent with the IRL view.

### 2.1 Protein language models for fitness prediction

#### 2.1.1 Definition

The protein fitness landscape describes how a protein’s function changes with its sequence, which can be quantitatively measured by methods like DMS (Fowler and Fields 2014). In DMS, fitness is a quantitative measure of a protein variant’s functional performance under specific selective pressure. Fitness *F* is calculated as the relative change in a variant’s abundance $N^{\text{mt}}$ from the pre-selection to the post-selection population, normalized to the change in the wild-type’s abundance $N^{\text{wt}}$:


(1) $$F(S^{\text{mt}},S^{\text{wt}})=\text{log}\;\left(\frac{N_{\text{post}}^{\text{mt}}/N_{\text{pre}}^{\text{mt}}}{N_{\text{post}}^{\text{wt}}/N_{\text{pre}}^{\text{wt}}}\right)$$

where a positive fitness value indicates a beneficial mutation, a negative value indicates a deleterious mutation, and a value near zero suggests a neutral effect on the protein’s function. The specific biological meaning of fitness score depends directly on the type of selective pressure applied.

#### 2.1.2 Notation and assumption

We focus on substitutions and, consistent with common practice, assume that a small number of substitutions do not alter the protein’s backbone structure (Li *et al.* 2024a, 2024b, Su *et al.* 2024, Sun *et al.* 2024, Tan *et al.* 2024, Zhang *et al.* 2024, Tan *et al.* 2025). Given a wild-type protein with sequence $S^{\text{wt}}$ and structure $X^{\text{wt}}$, its mutant has a sequence $S^{\text{mt}}$ that differs from $S^{\text{wt}}$ at the mutation sites, while its backbone structure remains unchanged ($X^{\text{wt}}=X^{\text{mt}}$). The objective is to develop an unsupervised model that predicts the fitness score for each mutant, quantifying its functional change relative to the wild-type.

#### 2.1.3 Common practice

pLMs are trained on the MLM objective, learning to predict residues at masked positions based on the surrounding context (Lin *et al.* 2023, Wang *et al.* 2024). As detailed in Meier *et al.* (2021), this capability allows pLMs to score sequence variations by calculating the log-odds ratio between the mutant and wild-type proteins for a set of mutations M:


(2) $$L_{\text{MLM}}=-\sum_{i\in M}\;\text{log}\;P(s_{i}|S_{\backslash M})$$

(3) $$\begin{array}{cc} \hat{F}(S^{\text{mt}},S^{\text{wt}})=\sum_{i\in M}[\text{log}\;P(s_{i}=s_{i}^{\text{mt}}|S_{\setminus M}) & \\ -\;\text{log}\;P(s_{i}=s_{i}^{\text{wt}}|S_{\setminus M})] & \end{array}$$

Here, $S_{\backslash M}$ denotes the input sequence with each mutated position in M masked. This scoring method assumes an additive model for multiple mutation sites. In the zero-shot setting, the model evaluates the sequence using a single forward pass.

### 2.2 Protein evolution as a Markov decision process

We formalize protein evolution as a Markov decision process (MDP) where the state space S consists of all possible protein sequences, the action space A represents point mutations acting on amino acid residues (with deterministic transition dynamics), the reward function R:S→R encodes selective pressure (not known *a priori*), and expert demonstrations D contain observed evolutionary trajectories of stable proteins under natural selection.

This simplified MDP formulation provides a setting for drawing an analogy between IRL and protein evolution. We explicitly adopt three simplifying assumptions: (1) Markovian property: Transition probabilities depend solely on the current sequence state, neglecting epistatic dependencies on historical mutations (Starr and Thornton 2016). (2) Stationary reward: Fitness landscapes are assumed time-invariant, though environmental shifts may alter selection pressures. (3) Expert optimality: Observed sequences are treated as optimal with respect to *R*, despite evolutionary constraints such as local optima, since the evolutionary traversed space may be limited compared to the vast protein sequence space.

Although based on simplifying assumptions, the MDP abstraction captures core dynamics of protein evolution. Crucially, it allows us to interpret natural selection as an expert policy $\pi^{*}$ that maximizes long-term fitness. Unlike standard reinforcement learning (RL), which finds an optimal policy to maximize rewards, IRL (Ng and Russell 2000) works backward, inferring the reward function that best explains expert trajectories. Specifically, Maximum Entropy IRL (MaxEnt IRL) (Ziebart *et al.* 2008) refines this by assuming expert actions follow a Boltzmann distribution proportional to expected reward.

The MLM training objective of pLMs aims to maximize the log-likelihood of sequences by learning to predict masked amino acids given their context (Equation (2)). MaxEnt IRL, in turn, models the probability of an expert trajectory ζ under a reward function $R_{\theta }$ as


(4) $$P_{\theta }(\zeta )=\frac{\;\text{exp}(R_{\theta }(\zeta ))}{Z_{\theta }},\;Z_{\theta }=\sum_{\zeta^{\prime }}\;\text{exp}\;(R_{\theta }(\zeta^{\prime }))$$

Here, $Z_{\theta }$ is the partition function that normalizes probabilities across all possible trajectories $\zeta^{\prime }$. Given a dataset of expert demonstrations D, the MaxEnt IRL log-likelihood is


(5) $$L_{\text{IRL}}(\theta )=\frac{1}{\left|D\right|}\sum_{\zeta \in D}\;\text{log}\;P_{\theta }(\zeta )=\frac{1}{\left|D\right|}\sum_{\zeta \in D}R_{\theta }(\zeta )-\;\text{log}\;Z_{\theta }$$

So maximizing $L_{\text{IRL}}$ selects the reward best explaining the trajectories and is equivalent to minimizing the MLM objective (Equation (2)). Under the MaxEnt–Boltzmann assumption (Equation (4)), $P_{\theta }(S)\propto \;\text{exp}(R_{\theta }(S))$, so the pLM’s log-probabilities provide an affine surrogate for the reward. Consequently, reward differences are proportional to log-probability differences; in particular


(6) $$\begin{array}{cc} \Delta R_{\theta }(S^{\text{mt}},S^{\text{wt}})=\sum_{i\in M}[\text{log}\;P_{\theta }(s_{i}^{\text{mt}}|S_{\backslash M}) & \\ -\;\text{log}\;P_{\theta }(s_{i}^{\text{wt}}|S_{\backslash M})] & \end{array}$$

Under this assumption, pLM log-probabilities approximate the reward up to an affine transformation. Viewing experimental fitness as a relative reward, Equation (6) provides a useful interpretation: the pLM log-odds in Equation (3) approximate the reward difference between the mutant and wild-type sequences, serving as a zero-shot predictor for fitness $F(S^{\text{mt}},S^{\text{wt}})$.

A common practice in protein fitness prediction is to supplement pLMs with evolutionary information from homologous sequences, which has been shown to further boost performance (Li *et al.* 2024a, Tan *et al.* 2024). Similarly, in large language models, a technique called *self-evolution* has emerged, where models use prior problem-solving trajectories as *context* to improve their reasoning and agentic abilities (Fu *et al.* 2025, Han *et al.* 2025, Wen *et al.* 2025, Zhao *et al.* 2025). This parallel suggests an intuitive explanation: just as humans learn from examples and adapt their reasoning based on relevant context, both pLMs and general language models can benefit from incorporating evolutionary trajectories as contextual demonstrations. In the protein domain, homologous sequences retrieved via sequence similarity searches (Edgar and Batzoglou 2006) or structure-based searches (van Kempen *et al.* 2024) provide evolutionary trajectories that act as expert demonstrations, constraining the solution space to biologically plausible mutations.

### 2.3 Sequence–structure model for fitness prediction

While pLMs are powerful for predicting mutational effects, incorporating 3D structural information has emerged as a common strategy to enhance their predictive performance (Su *et al.* 2024, Tan *et al.* 2024, Zhang *et al.* 2024). Our model builds upon S2F in Zhang *et al.* (2024) to enhance mutational effect prediction. We augment pLM features with geometric context by using a graph neural network (GNN) to process protein backbone structure. Specifically, we use geometric vector perceptron (GVP) (Jing *et al.* 2021) networks for message passing on a protein’s graph representation. The GVP module ensures SE(3)-invariance for scalar features and SE(3)-equivariance for vector features, which is crucial for handling 3D structural data. This architectural choice follows prior evaluations demonstrating GVP’s effectiveness for fitness prediction (Zhang *et al.* 2024), which we further validate in the supplementary architecture ablation comparing GVP with GearNet (Table S6, available as supplementary data at *Bioinformatics* online).

Formally, the hidden state of residue *i* at layer *l*, $h_{i}^{\left(l\right)}$, is represented by *d*-dim scalar features and $d^{\prime }$-dim vector features. Initial node features are set using ESM-2 embeddings, with $h_{i}^{\left(0\right)}=\left(\mathrm{ESM}-2\left(s_{i}|S_{\backslash M}\right),0\right)$. Edge features $e_{\left(j,i\right)}$ encode pairwise distances and coordinate differences using radial basis function (RBF) kernels. Message passing is performed using GVP modules, which process both scalar and vector features while ensuring SE(3)-invariance and SE(3)-equivariance, respectively. Each GVP layer is followed by a feed-forward network:


(7) $$\begin{array}{cc} & h_{i}^{\left(l+0.5\right)}=h_{i}^{\left(l\right)}+\frac{1}{\left|N(i)\right|}\sum_{j\in N(i)}\mathrm{GVP}\left(h_{j}^{\left(l\right)},e_{\left(j,i\right)}\right)\\ & h_{i}^{\left(l+1\right)}=h_{i}^{\left(l+0.5\right)}+\mathrm{GVP}\left(h_{i}^{\left(l+0.5\right)}\right) \end{array}$$

Finally, the scalar features from the last layer, $h_{i}^{\left(L\right)}$, are used to predict the residue type via a linear layer.

### 2.4 Evolutionary profiles for fitness prediction

#### 2.4.1 Sequence and structure profiles

MSA (Edgar and Batzoglou 2006) serves as a fundamental tool in computational protein modeling, capturing evolutionary relationships and co-evolutionary signals. While MSA-based approaches are widely applied to diverse tasks like protein structure prediction, function prediction, and design, and remain a mainstream strategy for protein fitness prediction, the raw MSA format poses practical challenges. Its variable length and depth, as well as potential alignment errors, may compromise both accuracy and efficiency in scaled models. As a result, recent research in protein design (Gong *et al.* 2025), structure prediction (Passaro *et al.* 2025), and optimization (Lv *et al.* 2025) has converged on using evolutionary profiles as a more compact and manageable evolutionary representation. For a protein with *n* aligned sequences $\left\{S_{1},S_{2},\ldots ,S_{n}\right\}$, each of length *L*, the evolutionary profile is represented as a matrix $P\in R^{L\times 21}$, where each entry $P_{ij}$ denotes the frequency of amino acid $A_{j}$ (including one special gap character “–”) at position *i* across the aligned sequences:


(8) $$P_{ij}=\frac{1}{n}\sum_{k=1}^{n}I\left(S_{k,i}=A_{j}\right)$$

Here, I(·) is the indicator function, $A_{j}\in A\cup \{-\}$ and *A* denotes the set of 20 standard amino acids. In addition to using sequence profiles, Tan *et al.* (2024) also constructs evolutionary profiles from structurally retrieved homologous sequences via Foldseek (van Kempen *et al.* 2024). Such structure profiles broaden the scope of this compact representation beyond pure sequence-based homology.

#### 2.4.2 IF profile

While evolutionary profiles are a powerful and compact representation of evolutionary information, their quality is directly dependent on the homologous search used to construct them. This process suffers from two primary limitations: (1) limited scope: the search often retrieves only the most closely related homologs, lacking coverage of the broader cross-family structural evolutionary landscape; (2) computational cost: searching massive databases for homologs is computationally expensive and time-consuming, often taking tens of minutes for a single protein. Given these limitations, we explore how to integrate evolutionary information more efficiently and comprehensively through IF profiles. Recent work (Shanker *et al.* 2024, Fei *et al.* 2025) shows that IF models trained on structure-conditioned sequence recovery tend to favor amino acid choices that mirror natural variation. Because they are trained on natural protein structures and sequences, they can capture the complex distribution patterns of protein sequences shaped by evolutionary dynamics. We therefore take the likelihood provided by IF models as an informative IF profile.

#### 2.4.3 Fusion module

To effectively integrate the complementary information from sequence–structure modeling and evolutionary profiles, we design a fusion strategy that processes each probability distribution through a transformer layer as transition block before combination. Given the S2F structural representation probabilities $P^{S2F}\in R^{L\times 21}$, evolutionary profile probabilities $P^{\text{struct}}\in R^{L\times 21}$, and IF profile probabilities $P^{\text{IF}}\in R^{L\times 21}$, where *L* is the sequence length, the model’s predicted logits is obtained by:


(9) $$\begin{array}{cc} P_{\text{final}}=\text{softmax}(P^{S2F}+\text{Transition}(P^{\text{struct}}) & \\ +\text{Transition}(P^{\text{IF}})) & \end{array}$$

This fusion strategy allows the model to capture contextual relationships within each probability distribution through the transition block, then combine the processed distributions through addition and normalize the result to ensure valid probability distributions.

### 2.5 Pre-training and inference

We adopt the pre-training and inference recipe outlined in Devlin *et al.* (2019) and Zhang *et al.* (2024). For pre-training, we employ the MLM objective on the non-redundant subset of the CATH v4.3.0 dataset (Sillitoe *et al.* 2021), comprising 30 ng the standard MLM loss (Equation (2)) with the fused probabilities in Equation (9), we obtain the loss function:


(10) $$L_{\text{MLM}}^{\text{fusion}}=-\sum_{i\in M}\;\text{log}\;P_{\;\text{final}}(s_{i}|S_{\backslash M})$$

where M represents the set of masked positions, $s_{i}$ is the true amino acid at position *i* from the training sequence, and $P_{\text{final}}$ is obtained from the multi-source fusion in Equation (9). The weights of the ESM-2 and ProteinMPNN models are frozen, with only the profile transition blocks for the external profiles and the GVP layers for the structure graphs remaining trainable. Comprehensive training details are provided in the supplementary material, available as supplementary data at *Bioinformatics* online.

During inference, fitness prediction follows the log-odds approach outlined in Equation (3), where the model calculates the log-odds ratio between mutant and wild-type sequences to estimate the functional impact of mutations. Specifically, for a mutant sequence $S^{\text{mt}}$ and wild-type sequence $S^{\text{wt}}$ with mutation sites M, the predicted fitness is computed as:


(11) $$\begin{array}{cc} \hat{F}(S^{\text{mt}},S^{\text{wt}})=\sum_{i\in M}[\text{log}\;P_{\text{final}}\left(s_{i}=s_{i}^{\text{mt}}|S_{\backslash M}\right) & \\ -\;\text{log}\;P_{\text{final}}\left(s_{i}=s_{i}^{\text{wt}}|S_{\backslash M}\right)] & \end{array}$$

where $P_{\text{final}}$ is the fused probability distribution from Equation (9), and $S_{\backslash M}$ denotes the input sequence with each mutated position in M masked.

We refer to this pre-training and inference setup as our base model, EvoIF (MSA-free). To enable fair comparisons with alignment-dependent baselines, we also report an MSA-enabled variant, EvoIF-MSA, following Zhang *et al.* (2024). EvoIF-MSA uses a simple *post hoc* ensemble strategy: EvoIF is combined at inference time with the MSA-only method GEMME (Laine *et al.* 2019) by summing standardized *z*-scores, following the ensemble evaluation convention used in S3F-MSA. This *post hoc* procedure does not modify the EvoIF architecture or its training protocol and is applied only when an MSA is available.

## 3 Experiments

### 3.1 Experimental settings

#### 3.1.1 Dataset

ProteinGym (Notin *et al.* 2023) is a widely-used benchmark for protein mutation effect prediction. It contains 217 DMS assays with over 2.5 million substitution mutations, covering key functional properties like stability, binding, and activity. The curated experimental DMS data provide standardized sequences, predicted structures, and evolutionary information for fair model comparison.

#### 3.1.2 Evaluation metrics

We employ five standard metrics: Spearman correlation, AUC, MCC, NDCG, and top-10% recall. All metrics are computed using standardized scripts from the ProteinGym repository. Detailed descriptions of all metrics are provided in the supplementary material, available as supplementary data at *Bioinformatics* online. Fitness values in ProteinGym are normalized as a preprocessing step, specifically centered and normalized to the interval [0, 1]. Additionally, fitness is inherently a normalized metric. Since our evaluation primarily uses Spearman correlation, which measures ranking accuracy, normalization does not affect our results, as rank-order relationships are invariant under monotonic transformations.

#### 3.1.3 Training setup

EvoIF is pre-trained on 30 948 non-redundant CATH v4.3.0 structures with 15% MLM masking, while ESM-2 and ProteinMPNN are frozen. We train only the GVP layers and profile transition blocks for 80 epochs on four NVIDIA H800 GPUs (approximately 5 h), using a mini-batch size of 32 per GPU, a learning rate of $1\times 10^{-3}$, and weight decay of 0.1.

Structural homolog profiles were generated with Foldseek (van Kempen *et al.* 2024) by searching structures converted from data/dompdb/against AlphaFold/Proteome (Varadi *et al.* 2022). The retrieval used an *E*-value cutoff of 10, a maximum of 1000 hits per query, sensitivity 9.5, and no coverage, sequence-identity, or minimum-alignment-length filtering. Full retrieval and A3M processing settings are provided in the supplementary material, available as supplementary data at *Bioinformatics* online.

#### 3.1.4 Comparison methods

We benchmark against a broad set of state-of-the-art unsupervised methods, categorized as follows; detailed descriptions of all methods are provided in the supplementary material, available as supplementary data at *Bioinformatics* online:

- Sequence-based models: ProGen2 XL (Nijkamp *et al.* 2023), CARP-640M (Yang *et al.* 2024), ESM-2-650M (Lin *et al.* 2023).
- Alignment-dependent models: DeepSequence (Frazer *et al.* 2021), MSA Transformer (Rao *et al.* 2021), Tranception L with retrieval (Notin *et al.* 2022a), EVE (Frazer *et al.* 2021), GEMME (Laine *et al.* 2019), TranceptEVE L (Notin *et al.* 2022b).
- IF models: ProteinMPNN (Dauparas *et al.* 2022), MIF (Yang *et al.* 2023), ESM-IF (Hsu *et al.* 2022).
- Sequence–structure hybrid models: MIF-ST (Yang *et al.* 2023), ProtSSN (Tan *et al.* 2025), SaProt (Su *et al.* 2024), S2F (Zhang *et al.* 2024), S3F (Zhang *et al.* 2024), ProSST (*K *= 2048) (Li *et al.* 2024b).
- Structure- and MSA-hybrid models: S2F-MSA (Zhang *et al.* 2024), S3F-MSA (Zhang *et al.* 2024), VenusREM (Tan *et al.* 2024), AIDO-Protein-RAG 16B (Li *et al.* 2024a, Sun *et al.* 2024).

### 3.2 Main results

Table 1 shows the results of our method and comparison methods. We observe that our method achieves superior or comparable performance across a wide range of baselines in different settings. EvoIF significantly outperforms sequence-based pLMs, MSA-based approaches, and IF models. This suggests that combining sequence–structure representations with evolutionary profile information can improve empirical fitness prediction performance in this benchmark. Within the MSA-free sequence–structure hybrid group, EvoIF performs competitively, exceeding S2F and S3F, which reports a higher Spearman correlation in Table 1. When further combined with MSA signals, EvoIF-MSA further improves performance and remains competitive with sequence–structure and structure–MSA hybrid models. It further demonstrates remarkable computational efficiency, with training over $10^{9}$ times faster than AIDO-Protein-RAG-16B and over 900 times faster than VenusREM (Fig. S2, available as supplementary data at *Bioinformatics* online).

**Table 1:** Overall results on ProteinGym benchmark.^a^

Model	Benchmark results					Model information				
	Spearman	AUC	MCC	NDCG	Recall	Seq.	Struct.	MSA	# Params.	# Data
ProGen2 XL	0.391	0.717	0.306	0.767	0.199	✓	✗	✗	6.4B	>1B
CARP	0.368	0.701	0.285	0.748	0.208				640M	41M
ESM-2	0.414	0.729	0.327	0.747	0.217				650M	49M
DeepSequence	0.419	0.729	0.328	0.776	0.226	✓	✗	✓	70M	N/A
MSA Transformer	0.434	0.738	0.340	0.779	0.224				100M	26M
Tranception L	0.434	0.739	0.341	0.779	0.220				700M	250M
EVE	0.439	0.741	0.342	0.783	0.230				240M	250M
GEMME	0.455	0.749	0.352	0.777	0.211				<1M	N/A
TranceptEVE L	0.456	0.751	0.356	0.786	0.230				940M	250M
ProteinMPNN	0.258	0.639	0.196	0.713	0.186	✗	✓	✗	2M	25K
MIF	0.383	0.706	0.294	0.743	0.216				3M	19K
ESM-IF	0.422	0.730	0.331	0.748	0.223				142M	19K
MIF-ST	0.383	0.717	0.310	0.765	0.226	✓	✓	✗	643M	19K
ProtSSN	0.442	0.743	0.351	0.764	0.226				148M	30K
SaProt	0.457	0.751	0.359	0.768	0.233				650M	40M
S2F	0.454	0.749	0.359	0.762	0.227				6M	30K
S3F	0.470	0.757	0.371	0.770	0.234				20M	30K
ProSST (*K *= 2048)	0.507	0.777	0.398	0.774	0.236				110M	18.8M
S2F-MSA	0.487	0.767	0.381	0.790	0.240	✓	✓	✓	246M	30K
S3F-MSA	0.496	0.771	0.387	0.792	0.244				260M	30K
VenusREM	0.518	0.783	0.404	0.770	0.244				110M	18.8M
AIDO-Protein-RAG	0.518	**0.784**	0.405	0.789	0.239				16B	1.2T
**EvoIF**	0.489	0.768	0.384	0.782	**0.250**	✓	✓	✗	76M	30K
**EvoIF-MSA**	**0.519**	**0.784**	**0.409**	**0.796**	0.246			✓	76M	30K

a Bold and underline indicate the best and second method for each metrics, respectively.

These results highlight both the effectiveness and efficiency of EvoIF and EvoIF-MSA. Our method enables much shorter training times than existing large-scale baselines and demonstrate strong capability in capturing evolutionary information.

### 3.3 Ablation study

#### 3.3.1 Profile type ablation

We evaluate the contribution of different profile types through systematic ablation studies (Table 2). Starting from a baseline model without any profile (Spearman correlation: 0.454), we observe that adding the IF profile alone improves performance to 0.478, while adding the evolutionary profile alone yields a smaller improvement to 0.462. The combination of both profiles achieves optimal performance (0.489), demonstrating their complementary nature and synergistic effect in capturing comprehensive biological information (Parameter counts refer to trainable parameters only. For methods using frozen pre-trained models (e.g. S2F, S3F, EvoIF use frozen ESM-2-650M and/or ProteinMPNN), only trainable components are counted.).

**Table 2:** Ablation of profile types on ProteinGym dataset.

Profile type		Metric				
Inverse folding	Structure	Spearman	AUC	MCC	NDCG	Recall
**✗**	**✗**	0.454	0.749	0.359	0.762	0.227
**✗**	**✓**	0.462	0.753	0.365	0.770	0.234
**✓**	**✗**	0.478	0.761	0.376	0.779	0.248
**✓**	**✓**	**0.489**	**0.768**	**0.384**	**0.782**	**0.250**

Bold indicates the best method for each metrics.

#### 3.3.2 Data ablation

We evaluate our model’s performance with varying training set sizes through random deletion to assess data efficiency. As shown in Fig. S1f, available as supplementary data at *Bioinformatics* online, reducing training data impacts performance, demonstrating that training data quantity remains crucial for protein fitness prediction. However, our method achieves competitive performance with only 30K samples compared to state-of-the-art methods that require 1.2 T training samples (AIDO-Protein-RAG-16B) or 18.8M samples (VenusREM). This efficiency stems from our model’s ability to effectively integrate evolutionary information from homologous constraints and structural constraints, enabling more efficient learning from limited data, with training time costs reduced by up to $10^{9}$-fold (Fig. S2, available as supplementary data at *Bioinformatics* online).

#### 3.3.3 Homology quantity ablation

As shown in Fig. S1e, available as supplementary data at *Bioinformatics* online, we evaluate the impact of homologous sequence quantity by progressively and randomly reducing the number of available sequences. The results indicate that model performance depends on the number of homologous sequences, although the effect is not pronounced. These findings demonstrate the importance of homologous sequence availability for protein fitness prediction. The results also demonstrate the capability of our method to maintain competitive performance even when homologous sequences are limited.

### 3.4 Analysis

Our method achieves superior performance across all tested scenarios, confirming that the structure-evolution joint representations are highly conserved and universal, with strong inductive biases that effectively compensate for limited evolutionary information, enabling accurate prediction of novel protein families. For a detailed qualitative analysis on a representative system, please refer to the case study in the supplementary material, available as supplementary data at *Bioinformatics* online. We observe consistent performance improvements as the model progressively incorporates multi-scale protein features. Figure S1a–d, available as supplementary data at *Bioinformatics* online, presents performance comparisons grouped by function type, MSA depth, taxon, and mutation depth:

#### 3.4.1 Function type

Our model demonstrates particularly strong performance in capturing organismal fitness and protein stability. For organismal fitness prediction, our method’s superior performance stems from its ability to capture evolutionary relationships between different organisms and distinguish functional constraints across species. For protein stability prediction, our model’s effectiveness arises from the direct relationship between protein structure and stability. While baseline methods (S2F, S2F-MSA) also incorporate structural information, our fundamental advantage lies in more comprehensive and efficient evolutionary encoding and representation capabilities, whereas sequence-based pLMs such as ESM-2 show clear limitations in capturing structure-related fitness effects.

#### 3.4.2 MSA depth

Sequence-only methods suffer from reduced performance at low MSA depths due to weak evolutionary signals. By contrast, our method provides a more efficient encoding of evolutionary information and achieves superior performance as MSA depth increases, effectively capturing conservation, co-variation, and mutational tolerance, while also retaining informative patterns in deep MSAs.

#### 3.4.3 Taxon

For underrepresented taxonomic group such as viruses, sequence-only models show reduced generalization capability due to taxonomic bias. This is because different viral families are often separated by larger evolutionary sequence distances. The sparsity of both known evolutionary sequences and experimental crystal structures for viruses contributes to this performance gap. However, our model still demonstrates performance improvements for viruses, indicating that efficient evolutionary encoding and structural inductive biases can compensate for insufficient data.

#### 3.4.4 Mutation depth

As the number of mutated sites increases, the performance of all methods declines due to the limitations of the additive mutation effect assumption. In contrast, our method remains more stable and outperforms other approaches at 2, 3, 4, and even ≥5 mutations, suggesting improved robustness for multi-mutant variants rather than explicit modeling of higher-order epistasis.

#### 3.4.5 Generalizing to novel protein families

While large-scale pLMs such as ESM-2 are pre-trained on massive sequence datasets like UniRef100, our methods (EvoIF and EvoIF-MSA) are trained on a much smaller dataset, using only 0.15% of the training data compared to large-scale models (Fig. S2, available as supplementary data at *Bioinformatics* online). A critical question arises: can the advantages of our methods generalize to protein families not seen during training? Figure S1g, available as supplementary data at *Bioinformatics* online, shows that in 23 out-of-distribution ProteinGym assays with low similarity to training data, all models exhibit performance degradation. However, our EvoIF and EvoIF-MSA methods consistently and significantly outperform the sequence-only baseline ESM-2. Moreover, our models also show a remarkable improvement over other baselines, demonstrating a superior ability to integrate both evolutionary profiles from homolog retrieval and IF profiles for more accurate predictions. Detailed out-of-distribution evaluation results are provided in the supplementary material, available as supplementary data at *Bioinformatics* online.

## 4 Discussion and conclusion

In this article, we introduce EvoIF, a lightweight and data-efficient framework for protein fitness prediction that combines an interpretive IRL view of pLM zero-shot scoring with a compact integration of evolutionary profiles and IF profiles. Extensive evaluation on ProteinGym demonstrates that EvoIF and its MSA-enabled variant EvoIF-MSA achieve competitive performance across 217 DMS assays while using only a fraction of the training data and parameters required by recent large-scale models. Ablations verify that the two profile sources are complementary, improving robustness across function types, MSA depths, taxa, and mutation depths.

EvoIF is also useful as a mutation-prioritization model for variant-effect prediction and early protein-engineering triage. Given a wild-type sequence and structure, it scores candidate substitutions and ranks variants for downstream validation when experimental throughput is limited. This use case applies to mutation-library prioritization, stability screening, enzyme or binding optimization, viral proteins, and engineered scaffolds with shallow or unreliable MSAs. Our function type, MSA depth, taxon, mutation depth, out-of-distribution, and ranking analyses provide application-oriented evidence for these settings.

This work highlights three takeaways. First, viewing MLM pre-training through the lens of IRL offers an interpretation of why pLM log-odds correlate with fitness under the assumptions discussed above. Second, a compact evolutionary representation that combines sequence- and structure-retrieved homolog profiles with IF profiles provides strong and uniformly available signals, mitigating the limitations of homolog searches in terms of limited scope and high computational cost. Third, a simple fusion via transition blocks suffices to yield calibrated probabilities for accurate log-odds estimation, obviating heavy model scaling.

Limitations include the absence of wet-lab validation, potential biases from structure availability and the site-additive nature of the log-odds score, which does not explicitly model higher-order epistasis. Future work will incorporate side-chain modeling and explore joint training of sequence–structure backbones with profile encoders. Diffusion-based design priors and inference-time retrieval adaptation are promising directions for enhanced generalization.

## Supplementary Material

btag525_Supplementary_Data

## Data Availability

All datasets used in this work are publicly available. Code is archived on Zenodo at https://doi.org/10.5281/zenodo.20139484.
