# Supplementary material for: Evolutionary profiles for protein fitness prediction
Source: Bioinformatics. 2026 Jul 20;42(8):btag525. doi: 10.1093/bioinformatics/btag525 (PMC13430659; doi:10.1093/bioinformatics/btag525)
Supplement: btag525_Supplementary_Data [file btag525_supplementary_data.pdf]

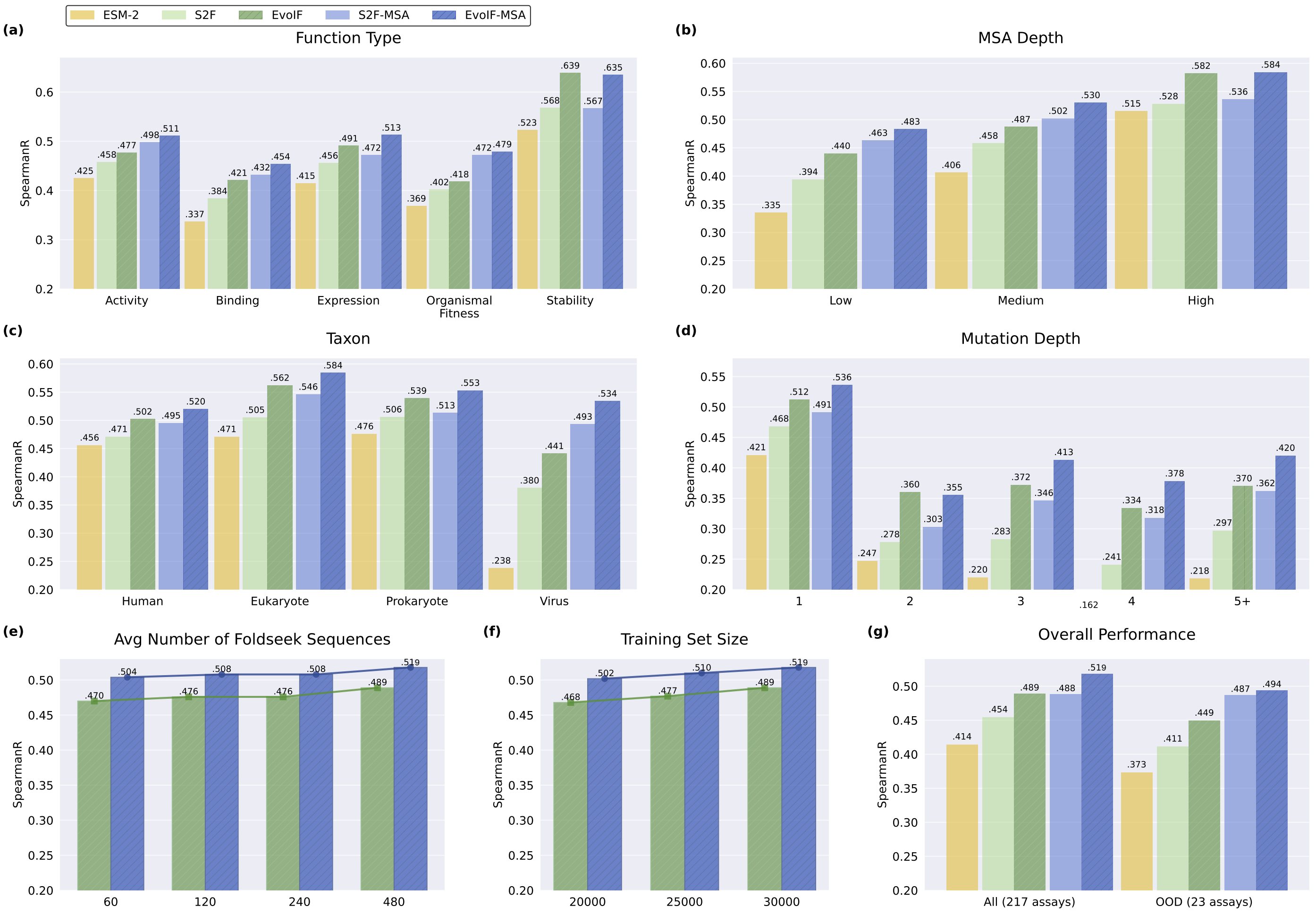

Figure S1 Breakdown analysis on ProteinGym, across **(a)** function type, **(b)** MSA depth, **(c)** taxon, and **(d)** mutation depth. **Ablation study** on **(e)** homology quantity and **(f)** training data size. **(g) Overall performance** on all assays and out-of-distribution assays.

Impact of Model and Data Scale on Protein Fitness Prediction Performance

We summarize how accuracy (Spearman) varies with model parameter count and pre-training data scale. As shown in Figure S2, scaling parameters or data yields limited marginal gains for protein fitness prediction relative to computational cost, which aligns with our design that emphasizes compact evolutionary representations and efficient fusion in EvoIF-MSA.

Notably, as illustrated in Figure S2, increasing model parameter count or pre-training data scale leads to only marginal improvements in protein fitness prediction performance relative to the associated computational cost. This is consistent with our design, which emphasizes compact evolutionary representations and efficient fusion mechanisms in EvoIF-MSA.

Case Study

Predicting the fitness of viral proteins is an important scientific problem. It enables the early identification of potential epidemiologically advantageous variants and accelerates the development of precise therapeutic strategies. In addition, accurate fitness prediction is highly valuable for engineering beneficial viruses such as bacteriophages. However, since different viruses are often separated by large evolutionary distances, the available within-family evolutionary information for viral proteins is usually limited. As a result, predicting the fitness of viral proteins has long been a challenge, and existing methods have struggled to achieve strong performance.

By explicitly modeling cross-family evolutionary information, our model achieves a significant improvement in viral fitness prediction (Figure S1). We select the Spike glycoprotein as a case study for analysis. This protein is essential for host cell recognition and membrane fusion and represents a central target for vaccine design and antibody neutralization. We compare our method with several baselines: the Spearman correlation coefficients of the sequence-based ESM2-650M model, the structure-based S2F-MSA model, and the evolution-based EvoIF-MSA model are -0.018, 0.366, and 0.559, respectively. These results demonstrate that EvoIF-MSA provides substantially more accurate fitness prediction. We further analyze the Spearman correlation coefficients of fitness prediction for different mutants at individual sites (Figure S3). EvoIF-MSA is able to better capture the mutational effects that are structurally close but lack sufficient within-family evolutionary information. This highlights

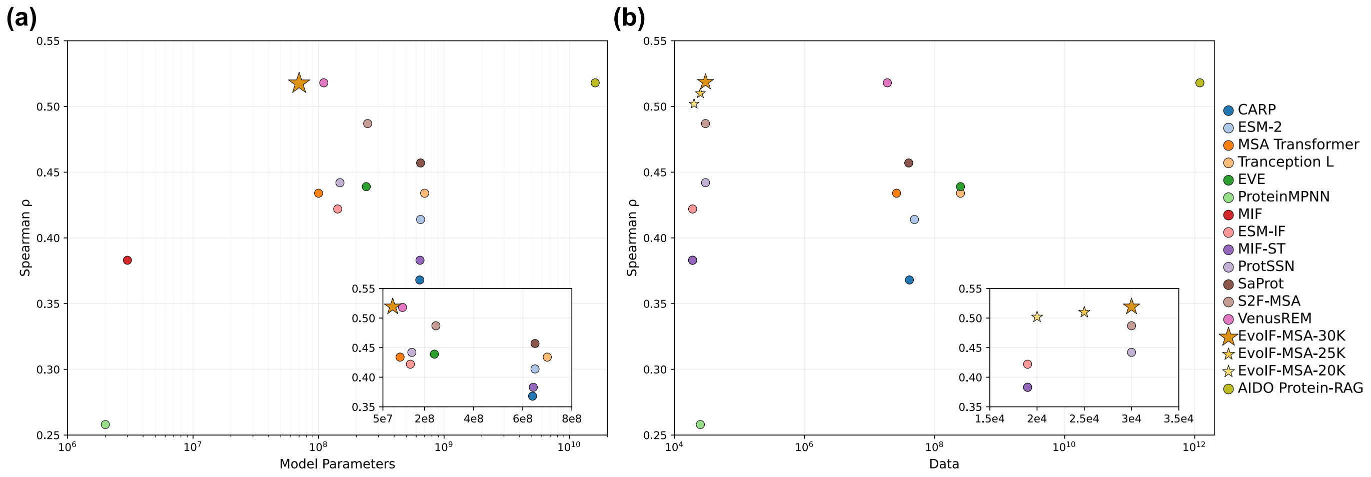

Figure S2 Accuracy (Spearman) versus **(a)** model parameters and **(b)** training data scale.

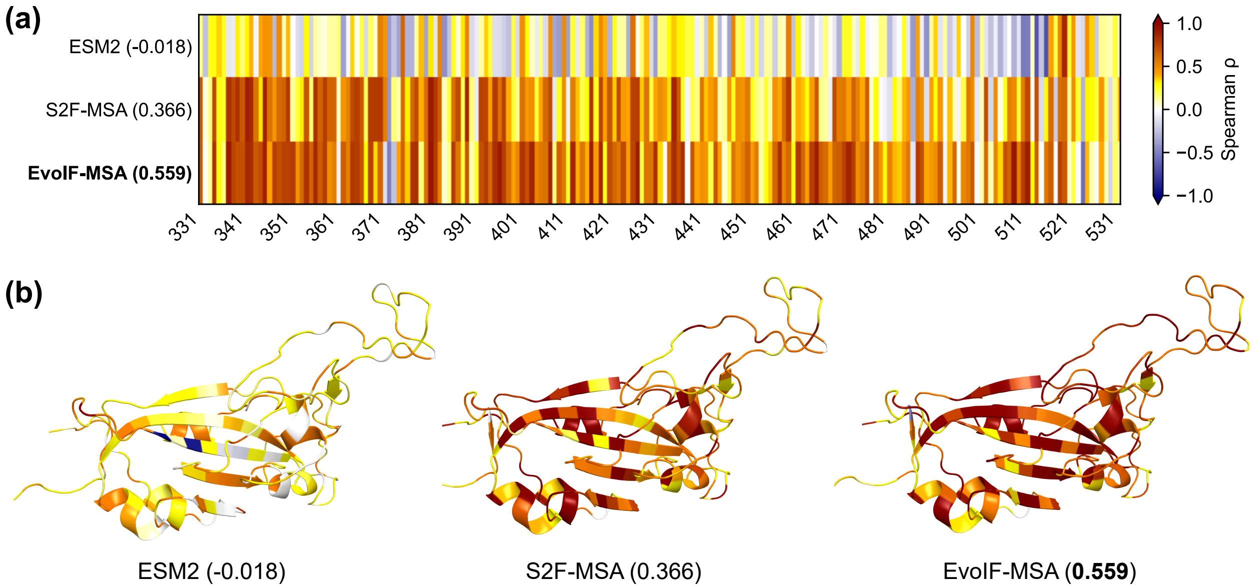

Figure S3 Visualization of fitness prediction results for the Spike glycoprotein. **(a)** Heatmap of per-site Spearman correlation coefficients of fitness prediction by ESM2-650M, S2F-MSA, and EvoIF-MSA. **(b)** Three-dimensional structure colored by per-site Spearman correlation coefficients of fitness prediction from ESM2-650M, S2F-MSA, and EvoIF-MSA. The structure was obtained from the ProteinGym database.

the advantage of EvoIF-MSA in providing a more comprehensive evolutionary profile for viral proteins.

Supplementary Related Work

Inverse Reinforcement Learning

Inverse Reinforcement Learning (IRL) infers a reward function from expert demonstrations rather than optimizing actions for a given reward. In Maximum Entropy IRL, expert behavior is modeled by a Boltzmann distribution over trajectories proportional to cumulative reward [Ng and Russell, 2000, Ziebart et al., 2008]. Viewing protein evolution as a sequential decision process, natural selection acts as the expert that preferentially retains high-fitness sequences. Under this lens, MLM on extant sequences resembles IRL: maximizing conditional log-likelihood aligns with maximizing an IRL objective on the expert's stationary distribution.

This correspondence implies that pLM log-probabilities provide an affine surrogate for reward; differences in log-probabilities (i.e., log-odds) approximate reward differences between mutant and wild-type, explaining the empirical success of zero-shot scoring used throughout the literature [Meier et al., 2021, Notin et al., 2023]. Extending the analogy, incorporating homologous sequences—retrieved by sequence or structure similarity—can be interpreted as supplying additional expert demonstrations *in context*, sharpening reward inference for the local family neighborhood. This perspective provides a principled rationale for combining pLMs with evolutionary context and motivates EvoIF's use of both homolog profiles and inverse folding priors for calibrated log-odds estimation.

Evolutionary Information Representation

Compact representations of evolutionary constraints have progressed from raw MSAs to profile-style and structure-aware surrogates. Classical alignment-based models use position-specific frequencies and co-evolutionary couplings derived from MSAs [Edgar and Batzoglou, 2006], but performance depends on family depth and retrieval quality. To improve scalability and uniformity, recent work in design and structure prediction emphasizes evolutionary profiles that summarize homolog statistics while remaining model-friendly [Gong et al., 2025, Passaro et al., 2025, Lv et al., 2025]. Structure-centric retrieval (e.g., Foldseek) expands beyond sequence-detectable homology, stabilizing profiles in remote regimes [van Kempen et al., 2024, Tan et al., 2024].

Inverse folding offers a complementary, cross-family source of evolutionary signal: structure-conditioned sequence recovery models assign high likelihoods to amino acids consistent with natural variation, thereby distilling structural–evolutionary couplings learned from broad protein space [Shanker et al., 2024, Fei et al., 2025]. These likelihoods function as informative, uniformly available priors, particularly valuable when MSAs are shallow, uneven, or expensive to retrieve. EvoIF integrates both sources—structure-retrieved homolog profiles and inverse folding likelihood profiles—through a lightweight transition block that fuses probabilities from sequence–structure backbones with compact evolutionary profiles. This design yields calibrated log-odds scoring while avoiding the computational cost and non-uniformity of deep homolog searches.

Implementation Details

Training Details

During pre-training, we randomly select 15% of the residues in each protein sequence and apply the following token modification scheme: 80% of the selected residues are replaced with a `[MASK]` token, 10% are swapped with a random residue token, and the remaining 10% are left unchanged. The model is then tasked with predicting the original, unmodified residue.

The weights of the ESM-2-650M and ProteinMPNN models are frozen, with only the profile transition blocks for the external profiles and the GVP layers for the structure graphs remaining trainable. We train our model on four NVIDIA H800 GPUs for 80 epochs, which takes approximately 5 hours. Empirically, a mini-batch size of 32 per GPU (128 in total) yields better representation quality than 64 or 128 per GPU, so we keep this setting throughout our experiments.

Hyper-parameters

We employ a hybrid optimizer that combines Muon Liu et al. [2025] for matrix parameters and AdamW Loshchilov and Hutter [2019] for other parameters. Matrix parameters (defined as parameters with dimensionality $\geq$2D) are optimized using Muon with a learning rate of 1×10^{-3}, momentum of 0.95, 5 Newton-Schulz steps, and weight decay of 0.1. The remaining parameters use AdamW with $\beta_1 = 0.9$, $\beta_2 = 0.95$, $\epsilon = 1 \times 10^{-8}$, and weight decay of 0.1. Parameters are automatically routed based on dimensionality, with Muon learning rates scaled by matrix dimensions to ensure stable convergence.

Homology Retrieval

For the structural homolog profile, we used Foldseek v10.941cd33 [van Kempen et al., 2024] to search structures converted from `data/dompdb/` against AlphaFold/Proteome [Varadi et al., 2022]. Searches used backtrace alignment, an E-value cutoff of 10, a maximum of 1000 hits per query, and sensitivity 9.5. We disabled coverage filtering, sequence-identity filtering, and minimum-alignment-length filtering by setting their thresholds to 0. Other search settings followed the release environment: combined 3Di and amino-acid alignment, alignment mode 3, alignment output mode 0, one search iteration, prefilter mode 0, no query masking, and no practical cap on accepted or rejected prefilter hits. Retrieved alignments were exported as unfiltered A3M-format structural MSAs and unpacked as per-query A3M files. The resulting A3M alignments were processed by realigning all sequences to the query length through truncation or padding while preserving gap characters (`"-"`). We then construct the position-specific profile $\boldsymbol{P}$ directly from the aligned homologs following the profile definition in the Main Text and use it as the evolutionary prior in our fusion module.

Evaluation Metrics

To comprehensively evaluate the performance of protein fitness prediction, we employ a set of five metrics: (1) Spearman's rank correlation coefficient (**Spearman**), which quantifies the monotonic relationship between model-predicted fitness scores and experimentally measured values, effectively capturing ordinal agreement without assuming linearity. (2) The area under the receiver operating characteristic curve (**AUC**) assesses binary classification performance across varying discrimination thresholds. (3) Matthews correlation coefficient (**MCC**) evaluates classification quality in the presence of class imbalance, offering a balanced perspective on prediction accuracy. (4) Normalized discounted cumulative gain (**NDCG**) measures the model's capability to correctly rank highly functional variants. (5) Top-10% recall (**recall**) calculates the proportion of truly functional mutants identified within the top decile of model predictions. All metrics are computed using standardized scripts from the ProteinGym repository to ensure reproducibility and consistency with established benchmarks.

Model Architecture

Figure S4 illustrates the Geometric Sequence-Structure Encoder component of EvoIF. Specifically, ESM features are used to initialize the node features within the Geometric Sequence-Structure Encoder (GNN). Beyond the GVP-GNN architecture shown in the figure, EvoIF incorporates two types of evolutionary profiles: (1) Evolutionary Profile ($\boldsymbol{P}^{\text{struct}}$) derived from structural homologs retrieved via Foldseek, and (2) Inverse Folding Profile ($\boldsymbol{P}^{\text{IF}}$) obtained from ProteinMPNN. Both profiles are processed through separate transition blocks (transformer layers) and then combined with the GNN output via addition at the logits level, as detailed in Equation 9 in the Main Text.

Additional Results

Main results are under the official ProteinGym v1.0 zero-shot protocol (217 substitution assays, assay-level averaging, five metrics). SSEmb [Blaabjerg et al., 2024] reports an average Spearman correlation of 0.453 under a different protocol: the original 87-assay substitution benchmark with nine validation assays excluded and assay-level Spearman correlations averaged by UniProt ID. To enable a fair text-level comparison, we re-evaluated EvoIF under the same SSEmb-style

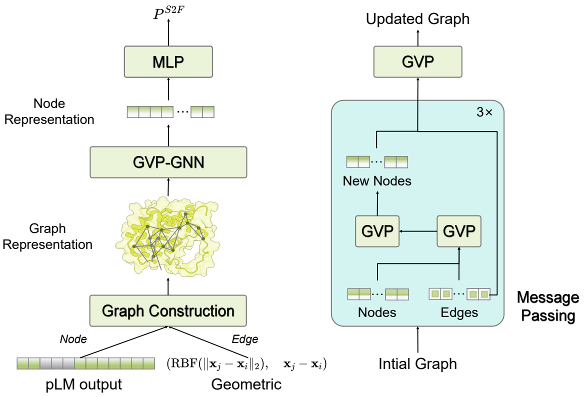

Figure S4 Geometric Sequence-Structure Encoder architecture of EvoIF.

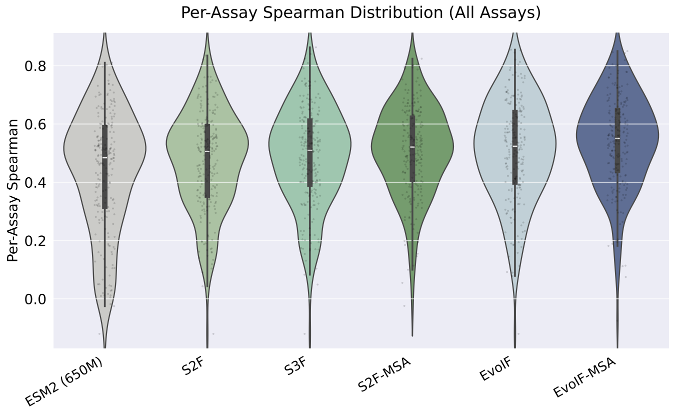

Figure S5 Distribution of per-assay Spearman correlations across all ProteinGym DMS substitution assays for ESM-2, S2F, EvoIF, S2F-MSA, and EvoIF-MSA.

protein-level protocol; under this matched protocol, EvoIF achieves a Spearman correlation of 0.458 (SSEmb: 0.453).

Additional Benchmarks

To evaluate EvoIF beyond the standard ProteinGym DMS assays, we considered ProteinGym Clinical Substitutions [Notin et al., 2023], which is closely related to ClinVar-like pathogenic/benign variant classification, and a MegaScale/Tsuboyama folding-stability subset [Tsuboyama et al., 2023].

Table S1 Results on the ProteinGym Clinical Substitutions benchmark.

Model	Avg. AUC
GEMME	0.919
S2F	0.877
EvoIF	0.856
ProteinMPNN	0.704

The clinical benchmark contains 2525 genes and reports average AUC across genes. GEMME achieved the highest average AUC, consistent with the strong role of sequence conservation and MSA-derived signals in pathogenic/benign variant classification. Among non-MSA baselines, S2F and EvoIF performed above the structure-only ProteinMPNN baseline.

Table S2 Results on the MegaScale/Tsuboyama folding-stability subset.

Model	Pearson
ProteinMPNN	0.5275
EvoIF	0.5306
S2F	0.5228

The MegaScale/Tsuboyama subset includes 298 domains and 271,231 single-substitution mutants with experimentally determined `ddG` labels and available AlphaFold backbones. We report Pearson correlation with experimentally determined `ddG`. EvoIF was comparable to ProteinMPNN and S2F under this metric, suggesting similar ranking behavior on this stability-oriented benchmark.

Additional Analyses

We present application-oriented analyses of EvoIF across protein function types and experimental conditions on the ProteinGym benchmark. These analyses connect benchmark performance to practical variant-effect prediction and protein-engineering workflows, where models rank candidate mutations before limited experimental validation.

Detailed Performance Across Function Types

We report the distribution of per-assay Spearman correlations across all DMS substitution assays (Figure S5) and per-assay Spearman correlations for activity assays (Figure S7), organismal fitness assays (Figure S8), stability assays (Figure S9), expression assays (Figure S10), and binding assays (Figure S11). These categories map to common engineering goals, including activity optimization, stability screening, expression improvement, and binding-affinity prioritization. Ranking metrics such as NDCG and top-10% recall are therefore especially relevant because they measure whether high-fitness variants are placed near the top of the candidate list for follow-up testing.

Out-of-Distribution Evaluation

Figure S6 shows the out-of-distribution evaluation results of EvoIF and EvoIF-MSA on 23 ProteinGym assays with low similarity to the training data. The results show that our approach consistently achieves superior performance under Out-of-distribution conditions, which highlights the strong generalization ability of EvoIF and EvoIF-MSA. The advantage is particularly evident for viral proteins, as they exhibit greater evolutionary heterogeneity. Viral families with similar functions often have low sequence similarity but share similar structural features. As a result, our explicit modeling of cross-family structural evolutionary information significantly improves the model's ability to capture comprehensive evolutionary signals. In addition, our method more effectively captures fitness effects across different mutation depths, which underscores its ability to model epistatic interactions associated with multiple mutations.

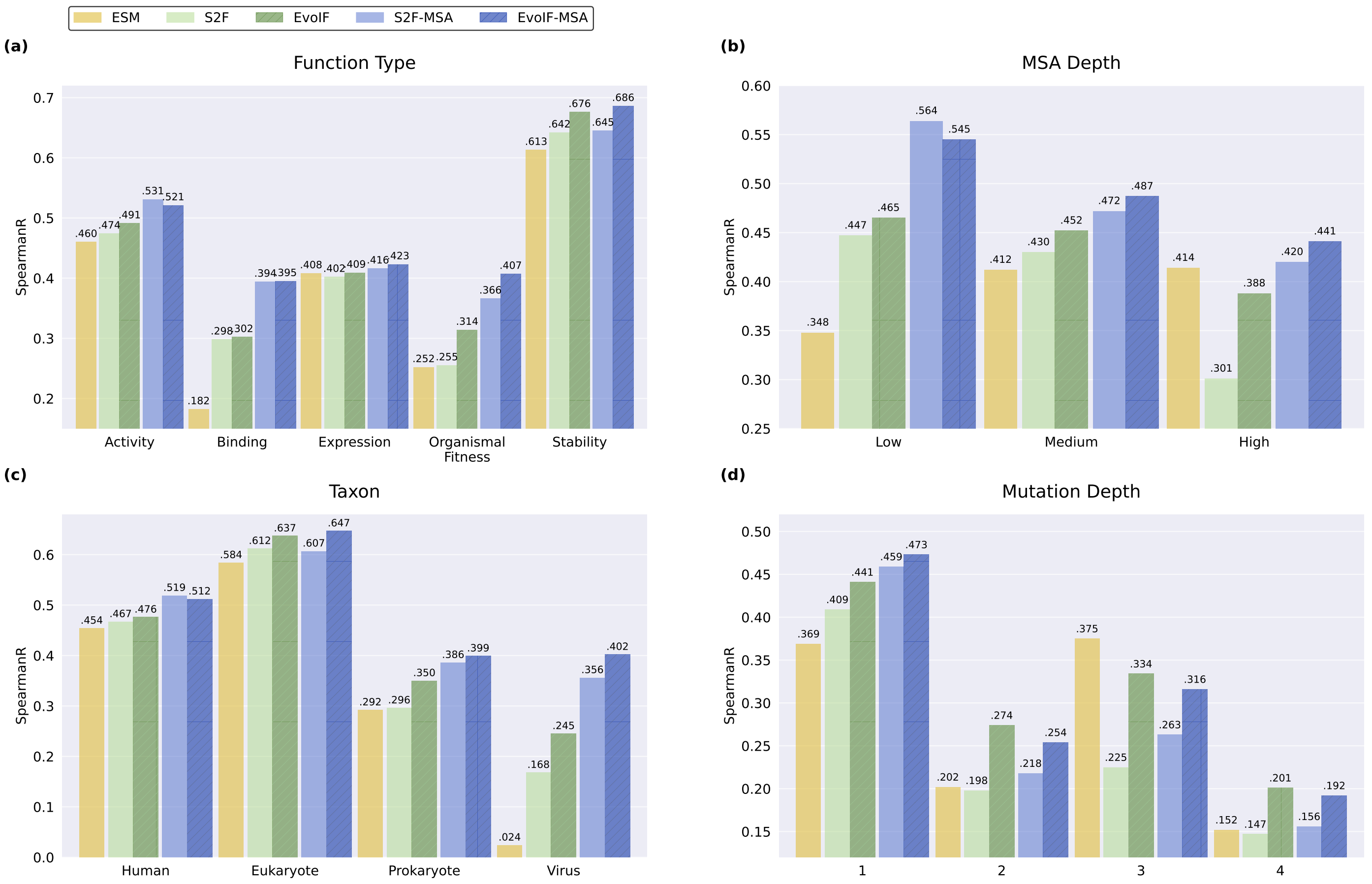

Figure S6 Out-of-distribution evaluation on 23 ProteinGym assays with low similarity to training data, across **(a)** Function Type, **(b)** MSA Depth, **(c)** Taxon, and **(d)** Mutation Depth. EvoIF and EvoIF-MSA maintain superior Spearman correlation compared to sequence-only and prior sequence–structure baselines.

Alternative Inverse Folding Models: ESM-IF and Caliby

To demonstrate that the effectiveness of inverse folding logits is not specific to ProteinMPNN, we evaluated our method using alternative inverse folding models: ESM-IF and Caliby. As shown in Table S3, all three inverse folding models (ProteinMPNN, ESM-IF, and Caliby) show consistent improvements when incorporating MSA ensemble, confirming that the benefits of using inverse folding logits stem from capturing evolutionary priors rather than being model-specific.

Table S3 Performance comparison across different inverse folding models (ProteinMPNN, ESM-IF, and Caliby) with and without MSA ensemble.

Inverse Folding Model	MSA	Spearman	AUC	MCC	NDCG	Top-recall
ProteinMPNN	✗	0.489	0.768	0.384	0.782	0.250
	✓	0.519	0.784	0.409	0.796	0.246
ESM-IF	✗	0.481	0.764	0.381	0.778	0.243
	✓	0.513	0.781	0.408	0.792	0.244
Caliby	✗	0.459	0.752	0.359	0.769	0.230
	✓	0.496	0.773	0.392	0.787	0.231

Impact of Homologous Sequence Similarity Threshold

To ensure that FoldSeek-retrieved homologs are within the same protein family and share similar evolutionary constraints, we conducted ablation studies varying the minimum sequence similarity threshold (0%, 20%, 30%, 40%, 50%). As shown in Table S4, model performance remains stable across different similarity thresholds, indicating that our method effectively utilizes structurally similar proteins while maintaining evolutionary relevance. The results demonstrate that FoldSeek's structural similarity search successfully identifies evolutionarily related proteins even at low sequence similarity levels.

Inference Time Analysis

We provide a comprehensive analysis of inference time for different components and methods. Table S5 reports the time required for FoldSeek homology search in the AlphaFold Database and ProteinMPNN inverse folding computation. Table S6 shows the MSA computation time for VenusREM on different proteins. Table S7 compares the total inference time across different methods on the ProteinGym benchmark, demonstrating that our method achieves competitive performance with reasonable computational overhead.

Table S4 Impact of homologous sequence similarity threshold on model performance. Results are reported for configurations with and without MSA ensemble.

MSA	Threshold	Spearman	AUC	MCC	NDCG	Top-recall
	0.0	0.519	0.784	0.409	0.796	0.246
	0.2	0.513	0.781	0.404	0.796	0.247
✓	0.3	0.515	0.782	0.406	0.793	0.244
	0.4	0.512	0.780	0.403	0.793	0.245
	0.5	0.510	0.780	0.401	0.792	0.242
	0.0	0.489	0.768	0.384	0.782	0.250
	0.2	0.482	0.764	0.379	0.785	0.246
✗	0.3	0.484	0.764	0.381	0.780	0.242
	0.4	0.481	0.763	0.378	0.777	0.241
	0.5	0.480	0.763	0.376	0.778	0.239

Table S5 Inference time for FoldSeek homology search in the AlphaFold Database and ProteinMPNN inverse folding computation.

Component	Dataset	# Proteins	Hardware	Inference Time
FoldSeek	CATH	30,948	64 CPU cores	33 min 45 sec
	ProteinGym	217	64 CPU cores	71 sec
ProteinMPNN	CATH	30,948	1 H800 GPU, 64 CPU cores	7 min 43 sec
	ProteinGym	217	1 H800 GPU, 64 CPU cores	10 sec

Table S6 MSA computation time for different proteins (96 CPUs). We selected several representative cases for analysis.

Protein	Sequence Length	Time
YNZC_BACSU	39	5h 18m
VKOR1_HUMAN	163	5h 1m
Q6wV13_9MAXI	222	4h 47m
C6KNH7_9INFA	566	5h 11m

Table S7 Total inference time comparison across different methods on the ProteinGym benchmark (excluding MSA recomputation time).

Method	Dataset	Inference Time
VenusREM	ProteinGym	3h 6m 36s
S2F	ProteinGym	1h 4m 58s
S3F	ProteinGym	6h 53m 48s
EvolF	ProteinGym	1h 12m 6s

Architecture Ablation: GVP vs GearNet

To validate our choice of GVP as the structure encoder, we conducted an ablation study comparing GVP with GearNet, another graph neural network architecture commonly used for protein structure modeling. As shown in Table S8, while both architectures benefit from incorporating MSA ensemble, GVP consistently outperforms GearNet across all metrics. This finding aligns with the evaluation reported in Zhang *et al.* [Zhang et al., 2024], confirming that GVP is more effective for fitness prediction tasks.

Table S8 Performance comparison between GVP and GearNet architectures with and without MSA ensemble.

Model	MSA	Spearman	AUC	MCC	NDCG	Top-recall
GVP	✗	0.489	0.768	0.384	0.782	0.250
	✓	0.519	0.784	0.409	0.796	0.246
GearNet	✗	0.473	0.758	0.371	0.771	0.237
	✓	0.508	0.777	0.397	0.792	0.242

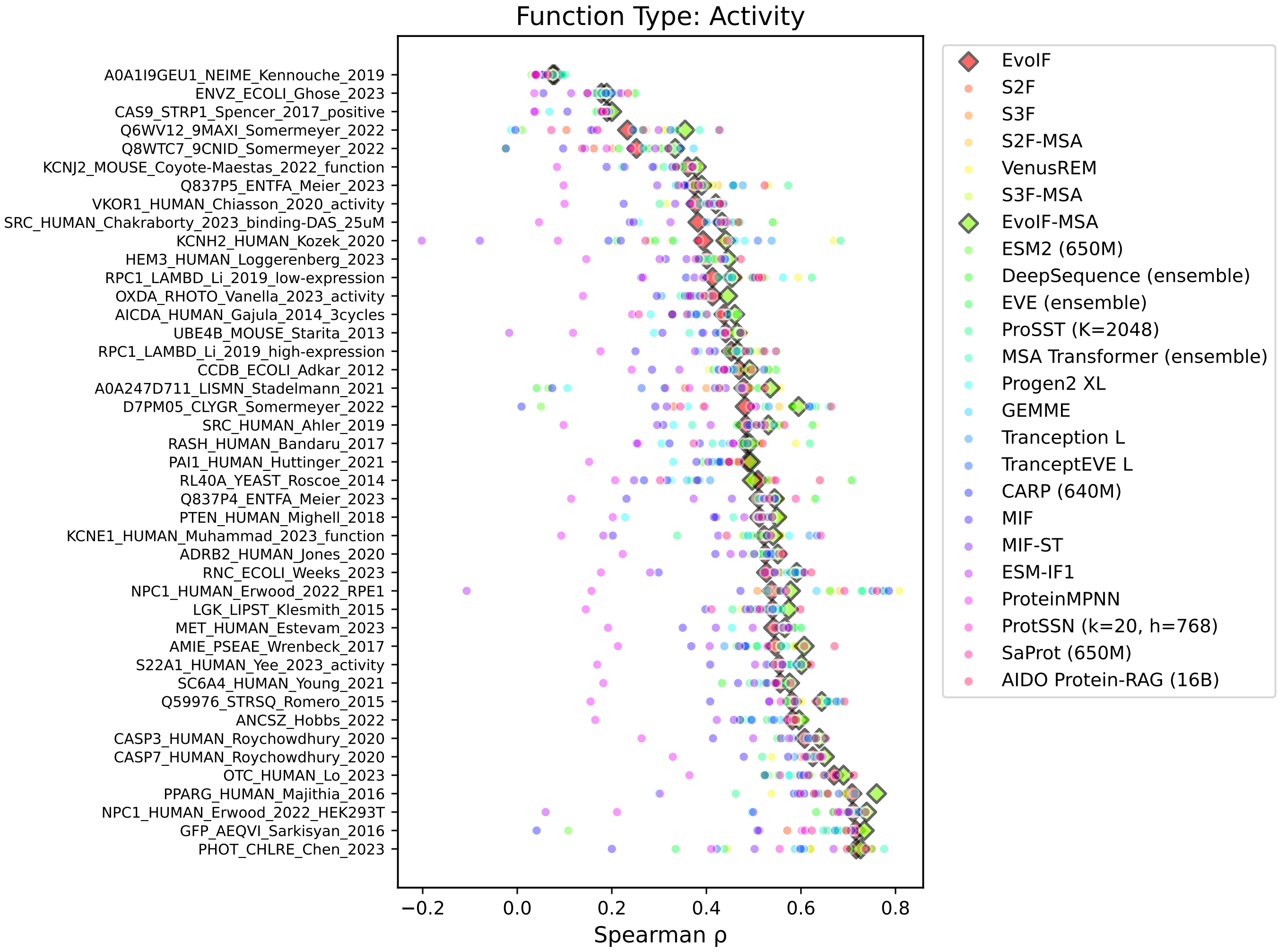

Figure S7 Per-assay Spearman correlation for activity assays on ProteinGym.

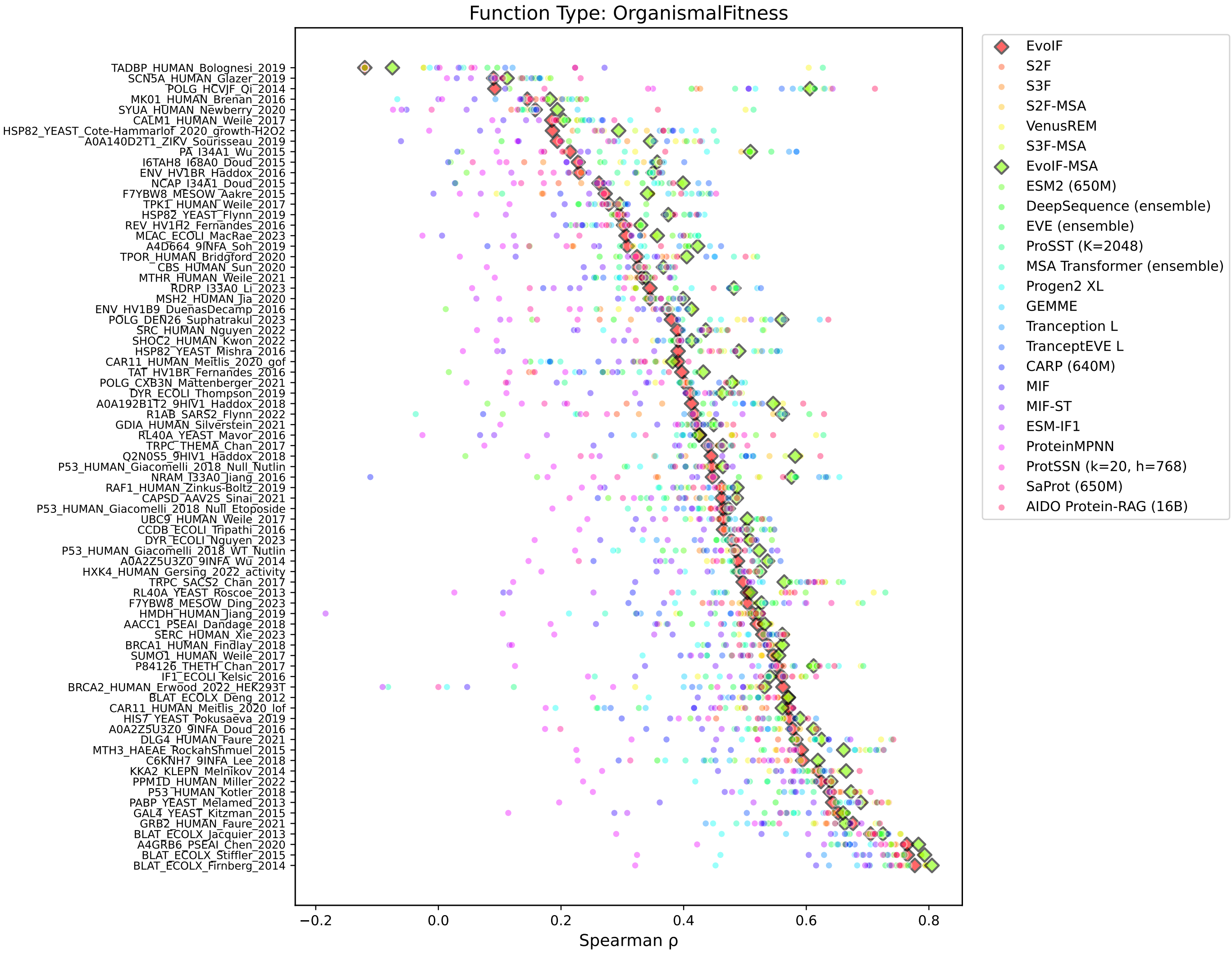

Figure S8 Per-assay Spearman correlation for organismal fitness assays on ProteinGym.

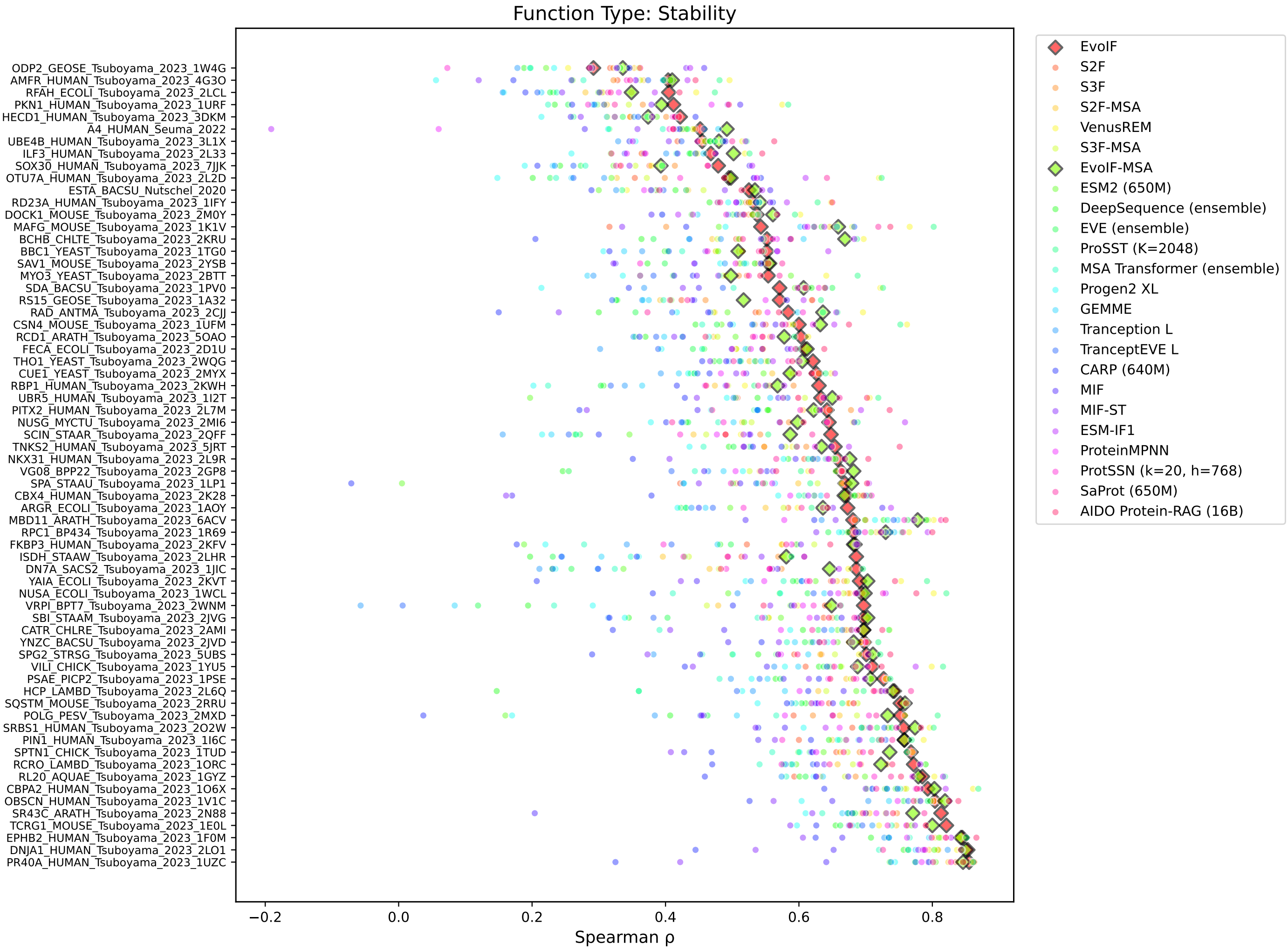

Figure S9 Per-assay Spearman correlation for stability assays on ProteinGym.

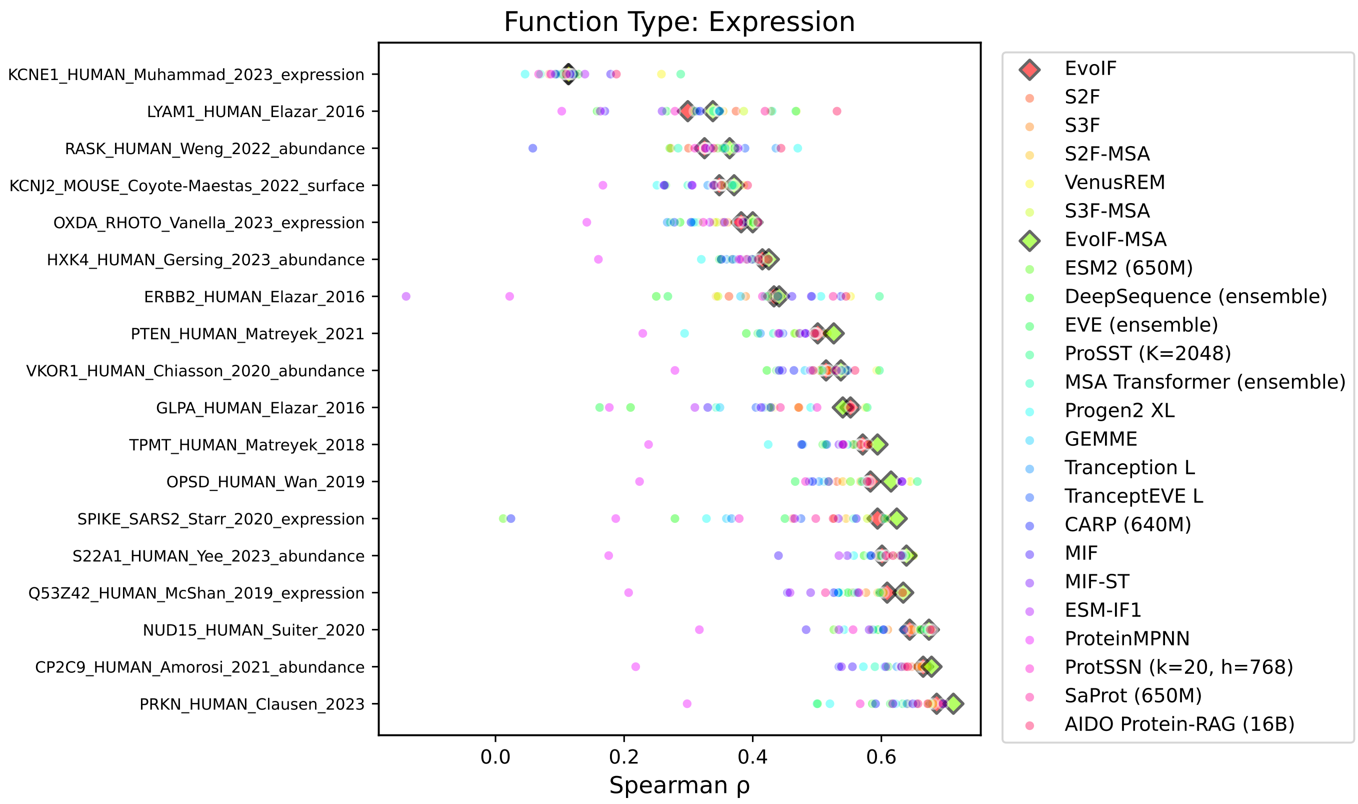

Figure S10 Per-assay Spearman correlation for expression assays on ProteinGym.

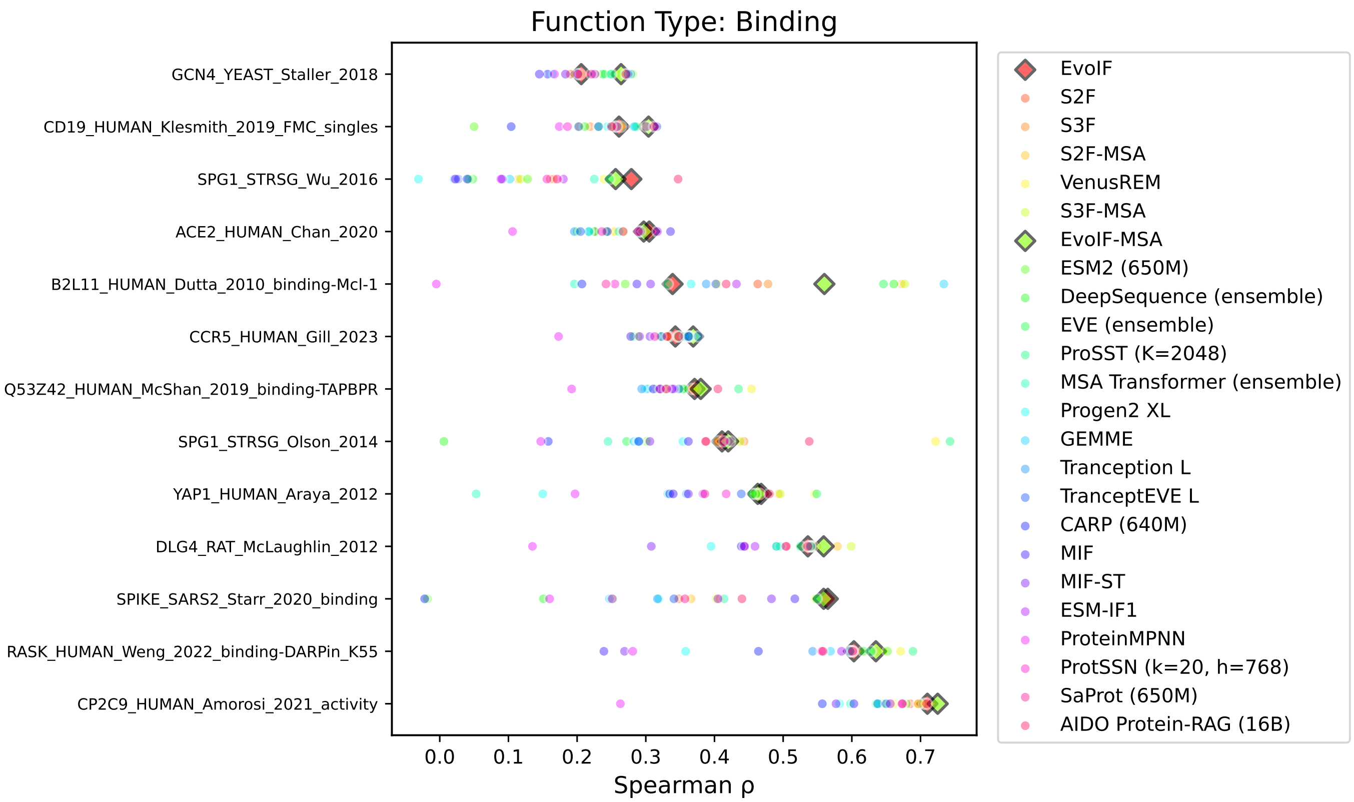

Figure S11 Per-assay Spearman correlation for binding assays on ProteinGym.

References

Andrew Y. Ng and Stuart J. Russell. Algorithms for inverse reinforcement learning. In *Proceedings of the Seventeenth International Conference on Machine Learning*, pages 663–670. Morgan Kaufmann, 2000.

Brian D. Ziebart, Andrew Maas, J. Andrew Bagnell, and Anind K. Dey. Maximum entropy inverse reinforcement learning. In *Proceedings of the Twenty-Third AAAI Conference on Artificial Intelligence*, pages 1433–1438. AAAI Press, 2008.

Joshua Meier, Roshan Rao, Robert Verkuil, Jason Liu, Tom Sercu, and Alex Rives. Language models enable zero-shot prediction of the effects of mutations on protein function. In *Advances in Neural Information Processing Systems*, volume 34, pages 29287–29303, 2021.

Pascal Notin, Aaron Kollasch, Daniel Ritter, Lood Van Niekerk, Steffanie Paul, Han Spinner, Nathan Rollins, Ada Shaw, Rose Orenbuch, Ruben Weitzman, Jonathan Frazer, Mafalda Dias, Dinko Franceschi, Yarin Gal, and Debora Marks. ProteinGym: Large-scale benchmarks for protein fitness prediction and design. In *Advances in Neural Information Processing Systems*, volume 36, pages 64331–64379, 2023. doi: 10.52202/075280-2810.

Robert C Edgar and Serafim Batzoglou. Multiple sequence alignment. *Current Opinion in Structural Biology*, 16(3): 368–373, 2006. doi: 10.1016/j.sbi.2006.04.004.

Jingjing Gong, Yu Pei, Siyu Long, Yuxuan Song, Zhe Zhang, Wenhao Huang, Ziyao Cao, Shuyi Zhang, Hao Zhou, and Wei-Ying Ma. Steering protein family design through profile bayesian flow. In *International Conference on Learning Representations*, 2025.

Saro Passaro, Gabriele Corso, Jeremy Wohlwend, Mateo Reveiz, Stephan Thaler, Vignesh Ram Somnath, Noah Getz, Tally Portnoi, Julien Roy, Hannes Stark, David Kwabi-Addo, Dominique Beaini, Tommi Jaakkola, and Regina Barzilay. Boltz-2: Towards accurate and efficient binding affinity prediction. *bioRxiv*, 2025. doi: 10.1101/2025.06.14.659707. URL https://www.biorxiv.org/content/10.1101/2025.06.14.659707.

Changze Lv, Jiang Zhou, Siyu Long, Lihao Wang, Jiangtao Feng, Dongyu Xue, Yu Pei, Hao Wang, Zherui Zhang, Yuchen Cai, Zhiqiang Gao, Ziyuan Ma, Jiakai Hu, Chaochen Gao, Jingjing Gong, Yuxuan Song, Shuyi Zhang, Xiaoqing Zheng, Deyi Xiong, Lei Bai, Wanli Ouyang, Ya-Qin Zhang, Wei-Ying Ma, Bowen Zhou, and Hao Zhou. AMix-1: A pathway to test-time scalable protein foundation model. *arXiv preprint arXiv:2507.08920*, 2025.

Michel van Kempen, Stephanie S. Kim, Charlotte Tumescheit, Milot Mirdita, Jeongjae Lee, Cameron L. M. Gilchrist, Johannes Söding, and Martin Steinegger. Fast and accurate protein structure search with Foldseek. *Nature Biotechnology*, 42(2): 243–246, 2024. doi: 10.1038/s41587-023-01773-0.

Yang Tan, Ruilin Wang, Banghao Wu, Liang Hong, and Bingxin Zhou. Retrieval-enhanced mutation mastery: Augmenting zero-shot prediction of protein language model. *arXiv preprint arXiv:2410.21127*, 2024.

Varun R. Shanker, Theodora U. J. Bruun, Brian L. Hie, and Peter S. Kim. Unsupervised evolution of protein and antibody complexes with a structure-informed language model. *Science*, 385(6704): 46–53, 2024. doi: 10.1126/science.adk8946.

Hongyuan Fei, Yunjia Li, Yijing Liu, Jingjing Wei, Aojie Chen, and Caixia Gao. Advancing protein evolution with inverse folding models integrating structural and evolutionary constraints. *Cell*, 188(17):4674–4692.e19, 2025. doi: 10.1016/j.cell.2025.06.014.

Jingyuan Liu, Jianlin Su, Xingcheng Yao, Zhejun Jiang, Guokun Lai, Yulun Du, Yidao Qin, Weixin Xu, Enzhe Lu, Junjie Yan, Yanru Chen, Huabin Zheng, Yibo Liu, Shaowei Liu, Bohong Yin, Weiran He, Han Zhu, Yuzhi Wang, Jianzhou Wang, Mengnan Dong, Zheng Zhang, Yongsheng Kang, Hao Zhang, Xinran Xu, Yutao Zhang, Yuxin Wu, Xinyu Zhou, and Zhilin Yang. Muon is scalable for LLM training. *arXiv preprint arXiv:2502.16982*, 2025.

Ilya Loshchilov and Frank Hutter. Decoupled weight decay regularization. In *International Conference on Learning Representations*, 2019.

Mihaly Varadi, Stephen Anyango, Mandar Deshpande, Sreenath Nair, Cindy Natassia, Galabina Yordanova, David Yuan, Oana Stroe, Gemma Wood, Agata Laydon, Augustin Žídek, Tim Green, Kathryn Tunyasuvunakool, Stig Petersen, John Jumper, Ellen Clancy, Richard Green, Ankur Vora, Mira Lutfi, Michael Figurnov, Andrew Cowie, Nicole Hobbs, Pushmeet Kohli, Gerard Kleywegt, Ewan Birney, Demis Hassabis, and Sameer Velankar. AlphaFold protein structure database: massively expanding the structural coverage of protein-sequence space with high-accuracy models. *Nucleic Acids Research*, 50(D1): D439–D444, 2022. doi: 10.1093/nar/gkab1061.

Lasse M. Blaabjerg, Nicolas Jonsson, Wouter Boomsma, Amelie Stein, and Kresten Lindorff-Larsen. SSEmb: A joint embedding of protein sequence and structure enables robust variant effect predictions. *Nature Communications*, 15(1):9646, 2024. doi: 10.1038/s41467-024-53982-z.

Kotaro Tsuboyama, Justas Dauparas, Jonathan Chen, Elodie Laine, Yasser Mohseni Behbahani, Jonathan J. Weinstein, Niall M. Mangan, Sergey Ovchinnikov, and Gabriel J. Rocklin. Mega-scale experimental analysis of protein folding stability in biology and design. *Nature*, 620(7973):434–444, 2023. doi: 10.1038/s41586-023-06328-6.

Zuobai Zhang, Pascal Notin, Yining Huang, Aurélie Lozano, Vijil Chenthamarakshan, Debora Marks, Payel Das, and Jian Tang. Multi-scale representation learning for protein fitness prediction. In *Advances in Neural Information Processing Systems*, pages 101456–101473, 2024. doi: 10.52202/079017-3217.
